# Supplementary material for: Machine Learning Accelerates Crystallization for Structure Determination
Source: Angew Chem Int Ed Engl. 2026 May 4;65(25):e1218503. doi: 10.1002/anie.1218503 (PMC13266958; doi:10.1002/anie.1218503)

checkCIF/PLATON report

Structure factors have been supplied for datablock(s) lcz079

THIS REPORT IS FOR GUIDANCE ONLY. IF USED AS PART OF A REVIEW PROCEDURE FOR PUBLICATION, IT SHOULD NOT REPLACE THE EXPERTISE OF AN EXPERIENCED CRYSTALLOGRAPHIC REFEREE.

No syntax errors found. CIF dictionary Interpreting this report

Datablock: lcz079

Bond precision:	C-C = 0.0092 A		Wavelength=1.54184
Cell:	a=9.3891(2)	b=25.8241(6)	c=13.8681(3)
	alpha=90	beta=90.059(2)	gamma=90
Temperature:	100 K		

	Calculated	Reported
Volume	3362.53(13)	3362.53(13)
Space group	P 21/c	P 1 21/c 1
Hall group	-P 2ybc	-P 2ybc
Moiety formula	C25 H14 Ag3 F18 N7 O3	C15 H3 Ag3 F18 N6, C10 H11 N O3
Sum formula	C25 H14 Ag3 F18 N7 O3	C25 H14 Ag3 F18 N7 O3
Mr	1126.04	1126.04
Dx,g cm-3	2.224	2.224
Z	4	4
Mu (mm-1)	15.220	15.220
F000	2160.0	2160.0
F000’	2170.52	
h,k,lmax	11,32,17	11,32,17
Nref	6924	6728
Tmin,Tmax	0.075,0.161	0.496,1.000
Tmin’	0.012	

Correction method= # Reported T Limits: Tmin=0.496 Tmax=1.000
AbsCorr = MULTI-SCAN

```
Data completeness= 0.972            Theta(max)= 74.972

                                                        wR2(reflections)=
R(reflections)= 0.0568( 4394)                           0.1394( 6728)

S = 1.081                   Npar= 507
```

The following ALERTS were generated. Each ALERT has the format
test-name_ALERT_alert-type_alert-level.
Click on the hyperlinks for more details of the test.

Alert level B

```
PLAT112_ALERT_2_B ADDSYM Detects New (Pseudo) Symm. Elem        C        96 %Fit
```

Author Response: The host has higher symmetry, but the co-crystallized molecules prevent the possible additional symmetry. The space group was checked with Platon, which indicated that there was pseudo-translation present, but that the correct P 21/c.

Alert level C

```
PLAT042_ALERT_1_C Calc. and Reported MoietyFormula Strings  Differ     Please Check
              Calc: C25 H14 Ag3 F18 N7 O3
              Rep.: C15 H3 Ag3 F18 N6, C10 H11 N O3
PLAT213_ALERT_2_C Atom F00K            has ADP max/min Ratio .....        3.4 prolat
PLAT213_ALERT_2_C Atom F00M            has ADP max/min Ratio .....        3.3 prolat
PLAT213_ALERT_2_C Atom F00Q            has ADP max/min Ratio .....        3.1 prolat
PLAT213_ALERT_2_C Atom F00R            has ADP max/min Ratio .....        3.6 prolat
PLAT213_ALERT_2_C Atom F00U            has ADP max/min Ratio .....        3.2 prolat
PLAT213_ALERT_2_C Atom F00W            has ADP max/min Ratio .....        3.6 prolat
PLAT213_ALERT_2_C Atom F011            has ADP max/min Ratio .....        3.3 prolat
PLAT220_ALERT_2_C NonSolvent   Resd 1  F   Ueq(max)/Ueq(min) Range        4.6 Ratio
PLAT250_ALERT_2_C Large U3/U1 Ratio for <U(i,j)> Tensor(Resd    1)        2.3 Note
PLAT342_ALERT_3_C Low Bond Precision on  C-C Bonds ..............     0.00915 Ang.
PLAT420_ALERT_2_C D-H Bond Without Acceptor  N00I     --H00I     .     Please Check
PLAT906_ALERT_3_C Large K Value in the Analysis of Variance ......     81.726 Check
PLAT906_ALERT_3_C Large K Value in the Analysis of Variance ......      8.925 Check
PLAT906_ALERT_3_C Large K Value in the Analysis of Variance ......      6.849 Check
PLAT906_ALERT_3_C Large K Value in the Analysis of Variance ......      4.593 Check
PLAT906_ALERT_3_C Large K Value in the Analysis of Variance ......      2.192 Check
PLAT911_ALERT_3_C Missing FCF Refl Between Thmin & STh/L=    0.600          5 Report
                8 17  6,   6 15  8,   6 17 10,   5 18 10,   7 10 11,
PLAT934_ALERT_3_C Number of (Iobs-Icalc)/Sigma(W) > 10 Outliers ..          1 Check
               10  7  8,
```

Alert level G

```
PLAT004_ALERT_5_G Polymeric Structure Found with Maximum Dimension          1 Info
PLAT007_ALERT_5_G Number of Unrefined Donor-H Atoms .............          1 Report
              H00I
PLAT083_ALERT_2_G SHELXL Second Parameter in WGHT  Unusually Large      27.50 Why ?
```

```
PLAT242_ALERT_2_G Low    'MainMol' Ueq as Compared to Neighbors of       C01B Check
PLAT242_ALERT_2_G Low    'MainMol' Ueq as Compared to Neighbors of       C01F Check
PLAT242_ALERT_2_G Low    'MainMol' Ueq as Compared to Neighbors of       C01G Check
PLAT242_ALERT_2_G Low    'MainMol' Ueq as Compared to Neighbors of       C01J Check
PLAT434_ALERT_2_G Short Inter HL..HL Contact F011     ..F016     .       2.79 Ang.
                                                   1+x,y,z  =      1_655 Check
PLAT720_ALERT_4_G Number of Unusual/Non-Standard Labels ..........         69 Note
              Ag01    Ag02    Ag03    F004    F005    F006    F007    F008
              F009    F00A    O00B    F00C    N00D    O00E    N00F    N00G
              N00H    N00I    H00I    F00K    F00L    F00M    N00N    N00O
              F00P    F00Q    F00R    C00S    C00T    H00T    F00U    C00V
              F00W    C00X    C00Y    C00Z    C010    H010    F011    C012
              C013    C014    C015    H015    F016    C017    H017    C018
              H018    C019    C01A    H01A    C01B    C01C    C01D    C01E
              C01F    C01G    C01H    H01H    C01I    H01B    H01C    H01D
              C01J    C01K    H01E    H01F    H01G
PLAT883_ALERT_1_G Absent Datum for _atom_sites_solution_primary ..     Please Do !
PLAT908_ALERT_2_G Max. Perc. Data with I > 2*s(I) per Res.Shell .      70.46% Note
PLAT912_ALERT_4_G Missing # of FCF Reflections Above STh/L=  0.600        189 Note
PLAT933_ALERT_2_G Number of HKL-OMIT Records in Embedded .res File          6 Note
                6 15  8,   7 10 11,   7 22  6,   8  9 11,   8 17  6,   9 18  5,
PLAT969_ALERT_5_G The 'Henn et al.' R-Factor-gap value ..........      4.303 Note
              Predicted wR2: Based on SigI**2  3.24 or SHELX Weight 12.89
PLAT978_ALERT_2_G Number C-C Bonds with Positive Residual Density.          1 Info
```

```
   0 ALERT level A = Most likely a serious problem - resolve or explain
   1 ALERT level B = A potentially serious problem, consider carefully
  19 ALERT level C = Check. Ensure it is not caused by an omission or oversight
  15 ALERT level G = General information/check it is not something unexpected

   2 ALERT type 1 CIF construction/syntax error, inconsistent or missing data
  20 ALERT type 2 Indicator that the structure model may be wrong or deficient
   8 ALERT type 3 Indicator that the structure quality may be low
   2 ALERT type 4 Improvement, methodology, query or suggestion
   3 ALERT type 5 Informative message, check
```

Validation response form

Please find below a validation response form (VRF) that can be filled in and pasted into your CIF.

```
# start Validation Reply Form
_vrf_PLAT042_lcz079
;
PROBLEM: Calc. and Reported MoietyFormula Strings  Differ     Please Check
RESPONSE: ...
;
_vrf_PLAT213_lcz079
;
PROBLEM: Atom F00K            has ADP max/min Ratio .....        3.4 prolat
RESPONSE: ...
;
_vrf_PLAT220_lcz079
;
PROBLEM: NonSolvent   Resd 1  F   Ueq(max)/Ueq(min) Range        4.6 Ratio
RESPONSE: ...
```

```
;
_vrf_PLAT250_lcz079
;
PROBLEM: Large U3/U1 Ratio for <U(i,j)> Tensor(Resd    1)        2.3 Note
RESPONSE: ...
;
_vrf_PLAT342_lcz079
;
PROBLEM: Low Bond Precision on  C-C Bonds ..............    0.00915 Ang.
RESPONSE: ...
;
_vrf_PLAT420_lcz079
;
PROBLEM: D-H Bond Without Acceptor  N00I     --H00I     .     Please Check
RESPONSE: ...
;
_vrf_PLAT906_lcz079
;
PROBLEM: Large K Value in the Analysis of Variance ......     81.726 Check
RESPONSE: ...
;
_vrf_PLAT911_lcz079
;
PROBLEM: Missing FCF Refl Between Thmin & STh/L=    0.600          5 Report
RESPONSE: ...
;
_vrf_PLAT934_lcz079
;
PROBLEM: Number of (Iobs-Icalc)/Sigma(W) > 10 Outliers ..          1 Check
RESPONSE: ...
;
# end Validation Reply Form
```

It is advisable to attempt to resolve as many as possible of the alerts in all categories. Often the minor alerts point to easily fixed oversights, errors and omissions in your CIF or refinement strategy, so attention to these fine details can be worthwhile. It is up to the individual to critically assess their own results and, if necessary, seek expert advice.

PLATON version of 17/09/2025; check.def file version of 17/09/2025

---

duplicate check

No duplication found

Datablock lcz079 - ellipsoid plot

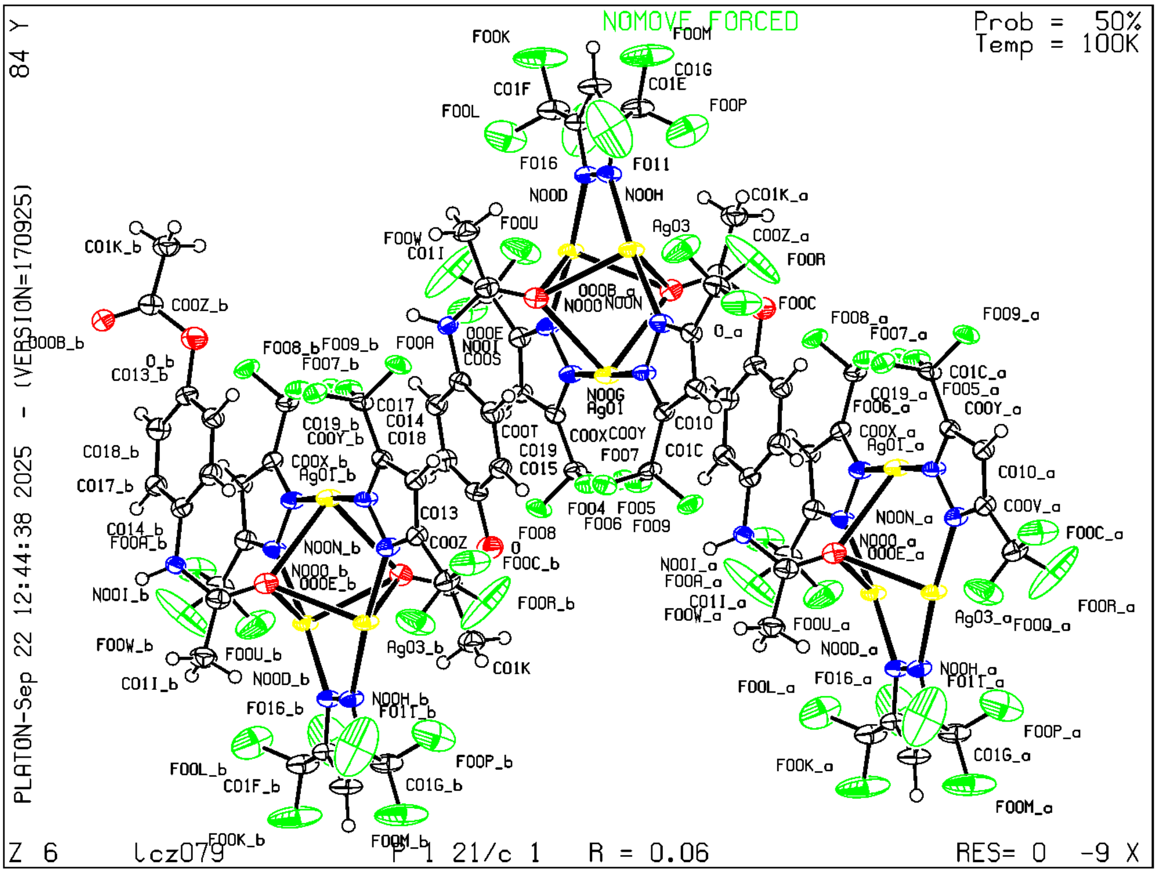

Supplement: Supplementary file 2 — Supporting File 2: anie72462‐sup‐0002‐DataFile.zip. [file ANIE-65-e1218503-s002.zip › supplementary_materials_crystallographic_data submit.zip!/cif_and_checkcif(1-114)/Ag3Pz3_79/Ag3Pz3_79_checkcif.pdf]
